# A Novel Approach for Indexing Heavy Metals Pollution to Assess Groundwater Quality for Drinking Purposes

**DOI:** 10.3390/ijerph17041245

**Published:** 2020-02-14

**Authors:** Elsiddig Eldaw, Tao Huang, Basheer Elubid, Adam Khalifa Mahamed, Yahaya Mahama

**Affiliations:** 1 Faculty of Geoscience and Environmental Engineering, Southwest Jiaotong University, Chengdu 611756, China; taohuang70@126.com (T.H.); elubaid@yahoo.com (B.E.); adamkh124@yahoo.com (A.K.M.); 2 College of Water and Environmental Engineering, Sudan University of Science and Technology, Khartoum 12304, Sudan; 3 School of Transportation and Logistics Engineering, Southwest Jiaotong University, Chengdu 611756, China; yahayamahama448@yahoo.com

**Keywords:** groundwater quality, heavy metals contamination, MHEI, NEI, PEI, SSMO

## Abstract

The present study proposes a new approach for indexing heavy metals ions to examine groundwater quality in North Kurdufan Province, Sudan. The new approach is developed based on the most frequently used methods for indexing heavy metals pollution in water. It is created in order to avoid the weaknesses of the current indexing systems. As per the new indexing approach, heavy metal contamination in water samples is evaluated by two types of indices: the negative evaluation index (NEI) and positive evaluation index (PEI). The water worthiness is assessed based on a pair of indices, NEI and PEI. Water quality increases with the decrease of PEI and NEI values. NEI indicates the contribution of heavy metals with a concentration not exceeding the highest desirable limit ($I_{i}$) in the water sample, while vice versa regarding the PEI. If all heavy metals concentrations in the water sample do not exceed $I_{i}$, the sum of NEI should be less than zero, but not less than −100, implying that the sum of PEI will be zero. When all heavy metals concentration exceeds $I_{i}$, the sum of NEI should be equal to zero, and PEI will be greater than zero. The results of the newly proposed approach have been discussed and compared with the existing indexing methods as regards to the best and worst samples. The spatial distribution of NEI and PEI are in complete agreement with the metals spatial distribution. The comparison result showed that the new index is robust, with fair calculations, and gives the best classification of groundwater quality.

## 1. Introduction

Groundwater has the advantage of low transmission cost, which makes it a perfect source for water supply, compared with surface water. Over the last two decades, the use of groundwater primarily for irrigation has increased significantly to meet the agricultural and economic development targets in many areas of Sudan, especially in regions far off the Nile system [1]. Unfortunately, these developments were implemented in an unplanned manner, which led to many problems for groundwater sources. In the study area, two sources of contaminants are expected to happen, which are considered to be an uncontrolled release. These groundwater contaminants sources come from: natural and anthropogenic (or man-made), that can alter the natural composition of groundwater. The natural source basically occurs from the rocks weathering, resulting from improper discharge from wells. The anthropogenic source includes municipal wastes, fertilizers, pesticides and many other factors that can reach to groundwater through groundwater recharge with surface water, which contain various pollutants [2]. While natural sources mainly include hazardous substances such as fluoride, nitrates, and heavy metals, which are found in the geological formations [2], once groundwater gets contaminated, it is difficult to reverse it to the pristine state [3].

Heavy metals contamination is posing a major problem to the aquatic environment and, therefore, are hazardous towards living beings [4,5]. Many prior studies concluded that there is an adverse health effect related to heavy metals exposure [6,7]. Water quality for the designated purpose is a function of water parameters concentration value with their desirable and permissible limits as per national and world health organization standards. Since the water sample contains a set of individual elements in different concentrations compared to the allowable limit for a particular purpose, it is difficult to judge water quality based on independent assessment. Thus, it is necessary to have a comprehensive quality assessment that will jointly take into account all the effects of water constituents. From this standpoint, the development of heavy metals pollution indexing methods (HPI${}_{s}$) is investigated. HPI${}_{s}$ are not limited to the aquatic environment but were applied to environment mold that comprises soil, sediment, foods, etc. [8,9,10,11,12].

Furthermore, the classification task is arduous as water quality does not only depend on the water constituent’s concentration but also on their relative importance in water as well as their toxicity. Therefore, the overall evaluation indices would make the task straightforward. Many researchers have developed different approaches to water quality indices and, understandably, all of them are semi-empirical [8,9,13,14,15,16,17,18,19]. Additionally, most of these techniques lack a theoretical basis, and their results vary quite a lot from each other. The methods mostly used for heavy metals indexing in groundwater quality assessment are proposed initially and formulated by Mohan, Edet and their co-authors [4,8,17,20,21] based on the highest desirable, maximum permissible and maximum allowable concentration. Also, Liu et al. [18,22] proposed an index to assess the environmental quality based on the maximum allowable concentration. These above methods have major drawbacks related to the estimation of the relative importance of individual elements in water quality, among others, which may lead to erroneous sample classification. In this work, a new approach is proposed to address these shortcomings while maintaining the advantage. We applied this approach to examine groundwater quality in north Kurdufan Province, Sudan, to assess the application potentials.

Past studies carried out for Kurdufan regions were limited to hydrological, geological, and hydrochemical properties of the confined and unconfined aquifers [23,24,25,26]. The studies concluded that the groundwater belonging to both aquifers inherited the physicochemical properties of sedimentary formations. In this research, we investigate groundwater quality and heavy metals risk based on the proposed modified heavy metal evaluation index (MHEI) in the case study of North Kurdufan Province, Sudan.

## 2. The Motivation for the New Approach

### 2.1. Heavy Metal Pollution Index (HPI)

HPI proposed by Mohan et al. [8], is used to determine overall water quality depending on heavy metal ions, and calculated according to Equation (1).

(1) $$HPI=\sum_{i=1}^{n}W_{i}Q_{i}$$

where $W_{i}$ is the relative weight or weighting factor for each chosen parameter defined as Equation (2); $Q_{i}$ is the individual quality rating for the ith heavy metal ion calculated for each parameter using Equation (3) and n is the number of parameters.

(2) $$W_{i}=\frac{w_{i}}{\sum_{i=1}^{n}w_{i}}$$

(3) $$Q_{i}=\frac{|M_{i}-I_{i}|}{\left(S_{i}-I_{i}\right)}\times 100$$

where $w_{i}$ means the unit weight factor for the ith heavy metal, which is inversely proportional to the maximum permissible value $S_{i}$ of the corresponding parameter as defined in Equation (4). $M_{i}$ is the measured concentration value of each parameter in the groundwater samples; $I_{i}$ and $S_{i}$ indicate the highest desirable and maximum permissible value standard of the ith parameters, respectively in according to Sudanese Standards and Metrology Organization (SSMO 2002), World Health Organization (WHO 2011), and (GB/T14848-1993) standards [27,28,29]. In the case of potable water, the maximum allowable value of HPI is 100, and less than 100 is considered suitable for drinking.

(4) $$w_{i}\propto \frac{1}{S_{i}}=\frac{k}{S_{i}}=\frac{1}{S_{i}}$$

where the proportionality factor *k* is taken equal to one for all metals in the literature [8,20].

### 2.2. Mean Metal Index (MI)

Tamasi and Cini [9] applied maximum allowed concentration (MAC) in estimating the value of MI and expressed by Equation (5).

(5) $$MI=\sum_{i=1}^{n}\frac{M_{i}}{MAC_{i}}$$

To assess the quality of drinking water, the value of MI can be divided into six classes: very pure (<0.3), purified (0.3–1.0), slightly affected (1.0–2.0), moderately affected (2.0–4.0), strongly affected (4.0–6.0) and seriously affected (>6.0) [10].

### 2.3. PoS Method

PoS index is developed by Tziritis et al. [18] to evaluate overall water quality depending on its physicochemical composition, and calculated according to Equation (6).

(6) $$PoS=\sum_{i=1}^{n}Q_{i}$$

(7) $$Q_{i}=\frac{\left(M_{i}\times W_{i}\right)}{S_{i}}\times 1000$$

where $Q_{i}$ is the individual quality contribution index, $M_{i}$ represents the monitored concentration of ith parameters, and $W_{i}$ is the relative weight for each chosen parameter defined as Equation (8).

(8) $$W_{i}=\frac{w_{i}}{\sum_{i=1}^{n}w_{i}}$$

where $w_{i}$ is the assigned weight value, which determines depend on its overall impact in terms of human toxicity. Based on the original PoS method [22], the partial scores of each parameter are allotted (Priority List of Hazardous Substances), as presented in Table 1.

According to the authentic PoS method, the groundwater samples are categorized into six classes indicating to water quality degradation level: minimum (1.0), low (2.0), medium (3.0), high (4.0), very high (5.0) and severe degradation (6.0) [18]. The reference index of PoS is calculated by supposing $M_{i}$ equating the maximum allowable limit (MAC${}_{i}$), which is subsequently used to determine the PoS categories. For more details on PoS index can be found in recent literature [30].

The formulations of the techniques mentioned above have some major shortcomings; we can summarize them in the following points:

1. The numerator term in Equation (3) can lead to the wrong classification. Consider the measured concentration values of zinc of two samples A and B to be 55 and 45 μg/L, respectively, and the highest desirable limit value of zinc is 50 μg/L; the effect of both concentrations will be the same, while in reality, sample A should fail, and sample B should pass the quality test.
2. As already known, a higher value of HPI indicates poor water quality and vice versa. Thus, when calculating the individual quality rating $Q_{i}$ using Equation (3), the $Q_{i}$ value adds to the overall index even when $M_{i}$ is less than $I_{i}$.
3. Also, the expression of MI and PoS are given in Equations (5) and (7). The influence of the sub-index for each heavy metal will be added to the overall index even when $M_{i}$ is less than MAC, leading to an erroneous increase of MI and PoS.
4. The estimation of MI as in Eqnuation (5) considered the concentration value of elements without regarding the toxicity to the overall water quality.
5. As per Equations (1) and (4), if the water sample encompasses many metals, the value of the relative weight of some elements such as zinc may be equal zero ($S_{i}$ = 3000). Thus, the influence of zinc metal in the water sample will be absent, even when $M_{i}$ is higher than $S_{i}$.
6. There are many rating ranges for HPIs as excellent, perfect, good, poor, and very poor regarding the water quality. However, the classifications of water quality by the aforementioned approaches are neither clear nor sufficient to determine the water quality. In theory and practice, the rating should be flexible depending on the level of influence of the individual concentration of elements as per water quality standard. This issue has been handled in developing the proposed MHEI method.

## 3. Materials and Methods

### 3.1. Sampling Site, Collection, and Analysis of Data

**Figure 1:**
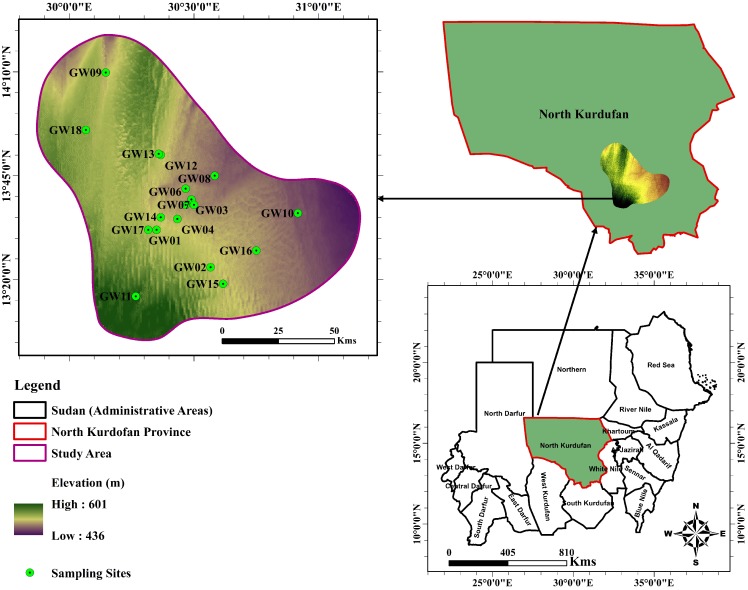
Map location of groundwater sampling stations of the study area.

The samples are collected from different locations in a populated area of North Kurdufan State. Figure 1 depicts the sampling sites. The geological formations in the area are combinations of (Pleistocene to Recent): (1) Basement complex of Precambrian, (2) Nawa Series (upper Paleozoic), (3) Nubian Series (Mesozoic) and (4) Um Ruwaba Series (Pliocene to Pleistocene) [23]. The samples are collected in one-liter polyethylene bottles after a thorough cleaning. The bottles were labeled before being transported and kept at a temperature below 4 °C until analyzed. Groundwater samples are filtered through a 0.45-μm Millipore membrane filter to separate the suspended sediments before analyzing them in the laboratory. The samples are collected from 18 groundwater extraction pump of a confined aquifer (Figure 1). The samples are collected after 10 min of pumping in one-liter polyethylene bottles after a thorough cleaning and repeated the samplings three once per well for each period (Three samples/well). The data are collected from January to December through two years 2017 and 2018; and statistically analyzed. It is noted that there was no significant variation through different periods in analyzing groundwater samples, so, the average value of groundwater quality parameters is considered in our study. The heavy metal ions are analyzed in quality-assured laboratories in Sudan (Environmental laboratory of the College of Water and Environmental Engineering, Sudan University of Science and Technology). The Inductively Couples Plasma—Optical Emission Spectrometry (ICP-OES) is applied to test concentration of heavy metals. Because it has been gaining favor with laboratories around the world as the instrument of choice for performing trace metal analysis. For accurate results, the ICP-OES was adjusted to generator parameter (Nebulizer Flow 0.81 L/min, Plasma Power 1300 W, Coolant Flow 15.0 L/min, and Auxiliary Flow 1.0 L/min), relative torch position (Horizontal 2.0, Vertical 6.5, and Distance 0.0), and measure time parameter (Total Time (s) 25.20, Netto Time (s) 24.00, and Stabilization Time (s) 0.00). The samples size to be tested should be at least 5 mL. Before analysis, we prepared standards for a range of concentrations consisting expected sample concentrations. The validation method experiment was carried out for the determination of metal content in the groundwater samples by Inductively Coupled Plasma Optical Emission Spectrometry (ICP-OES). The analytical measurements were made by using Spectro CIROS VISION ICP Model instrument. Data acquisition and processing were carried out using Smart Analyzer Vision software. The results of samplings analysis is obtained by interpolation method using linearity calibration curve for all parameters, with five (5) different concentrations. All the calibration coefficient of variations are better than 0.999. The limit of detection (LOD) and limit of quantitation (LOQ) of instrument for the parameters (mg/L) Fe, Mn, Zn, Pb, Cd, and Cr are 0.009 and 0.03, 0.13 and 0.44, 1.173 and 0.391, 0.039 and 0.13, 0.006 and 0.002, and 0.039 and 0.013, respectively. The estimation of expanded uncertainty of measurement (Spectro ICP) for six heavy metals μg/L are (Fe ± 0.6), (Mn ± 0.11), (Zn ± 0.14), (Pb ± 0.87), (Cd ± 0.70) and (Cr ± 0.12), with *k* = 2 for all metals. A preliminary assessment suggests that some elements are below the detection limit for all samples. Hence, the present study considered six heavy metal ions, namely iron (Fe), manganese (Mn), zinc (Zn), lead (Pb), cadmium (Cd), and chromium (Cr) in assessing groundwater quality. The parameters data obtained are compared with different standards for drinking purposes, including Sudanese Standards and Metrology Organization (SSMO 2002) [27], World Health Organization (WHO 2011) guidelines [28], and the Chinese Standard for Groundwater Quality (GB/T14848-1993) [29]. A global positioning system (GPS) has been used to determine the specific location of each sample. ArcGIS interpolation maps are used to reflect the spatial groundwater quality change pattern of the results of the prior indexing systems and the new approach.

### 3.2. Modified Heavy Metal Evaluation Index (MHEI)

In the present study, a new approach for water quality assessment is proposed, which extends the three most popular techniques in heavy metals indexing by the new elements as discussed below;

Supposing that the water sample consists of n number of heavy metal ions, then MHEI can be expressed by Equation (9).

(9) $$MHEI=\sum_{i=1}^{n}MHEI_{i}\sum_{i=1}^{n}\omega_{i}Q_{i}$$

where MHE$I_{i}$ is a modified heavy metal evaluation index of the ith indices, ω${}_{i}$ and $Q_{i}$ are the relative weight and a sub-index for the corresponding heavy metals and can be computed as in Equations (10) and (11), respectively.

(10) $$\omega_{i}=\frac{W_{i}}{\sum_{i=1}^{n}W_{i}}$$

(11) $$Q_{i}=\frac{\left(M_{i}-I_{i}\right)}{\left(S_{i}-I_{i}\right)}\times 100$$

where $W_{i}$ is the assigned weight value for each metal. We are considered the unit weights ($W_{i}$) of all heavy metals are inversely proportional to their corresponding MAC and defined as:

(12) $$W_{i}\propto \frac{1}{MAC_{i}}=\frac{k}{MAC_{i}}=\frac{1}{MAC_{i}}$$

where $M_{i}$ is the monitored concentration value of the ith parameters, and $S_{i}$ means maximum permissible concentration according to (SSMO 2002) and (GB/T14848-1993) standards. The constant of proportionality *k* is considered to be one for all metals. In this study, the (SSMO 2002) standard is considered. Where the specification is not available in (SSMO 2002) standards, the (GB/T14848-1993) standards guidelines are used. If the highest desirable value is not assigned in the standards, the maximum allowable value is used.

The new approach considers two indices to estimate MHEI; the negative evaluation index (NEI), and positive evaluation index (PEI) as defined below:

(13) $$NEI=\sum_{i=1}^{n1}MHEI_{i}$$

(14) $$PEI=\sum_{i=1}^{n2}MHEI_{i}$$

where *n*1 indicates the number of heavy metals whose $M_{i}$ value is equal or less than the highest desirable value, and *n*2 means the heavy metals with the $M_{i}$ value greater than the highest desirable value.

If the measured concentration value $M_{i}$ of a heavy metal ion in the water sample is equal to zero or below the detection limit (DL), the negative evaluation sub-index should be −100. If $M_{i}$ for all elements in the water samples is to equal 0 or ≤DL, the sum of negative evaluation indices NEI should be −100, implying the sum positive evaluation indices PEI for all heavy metal ions in the water sample will be zero. For measured value equal or less than the highest desirable limit ($M_{i}$ ≤ $I_{i}$), the PEI here equal to zero, whereas NEI is a negative number greater than −100. If the monitored value is less than the highest desirable limit, the NEI for the corresponding elements is zero; then, the PEI is a positive number strictly less than +100. If $M_{i}$ ≥ $S_{i}$ for all heavy metal concentrations in the water sample, the sum of positive evaluation indices PEI is equal or greater than +100, and the NEI will be zero. In the case of the water sample comprising sub-indices (PEI${}_{s}$ and NEI${}_{s}$) of different parameters, we add NEIs and PEIs separately to produce a pair of NEI and PEI indices as per Equations (13) and (14). Therefore, MHEI considers five levels in classifying water for drinking purpose, as listed in Table 2.

### 3.3. Spatial Interpolation Methods

The spatial interpolation is a numerical method that converts attribute database values of a set of locations (points) into a surface map that reflects the spatial change in these values throughout the study area. The spatial interpolation methods, for map generation, are a beneficial tool, which provides a piece of sensitive information for decision-makers. There are several mathematical methods to complete the spatial interpolation process, such as Kriging, Inverse Distance Weighted (IDW), Natural Neighbor, Spline, Trend, etc. However, the Kriging and IDW technique were widely used in environmental studies and the most appropriate interpolation tool to reflect the behavior of the movement of the contaminants inside the aquifer. In this study, we applied the IDW method to generate spatial interpolation maps.

#### Inverse Distance Weighted

IDW is a geo-statistical-based method, depend on the spatial engagement functions for investigation of the water parameters distribution within the study area. ArcGIS 10.3 software with the aid of (Spatial Analysis Tools) function is used to present the results of interpolation.

## 4. Results and Discussion

### 4.1. The Behavior of Heavy Metals in the Study Area

**Figure 2:**
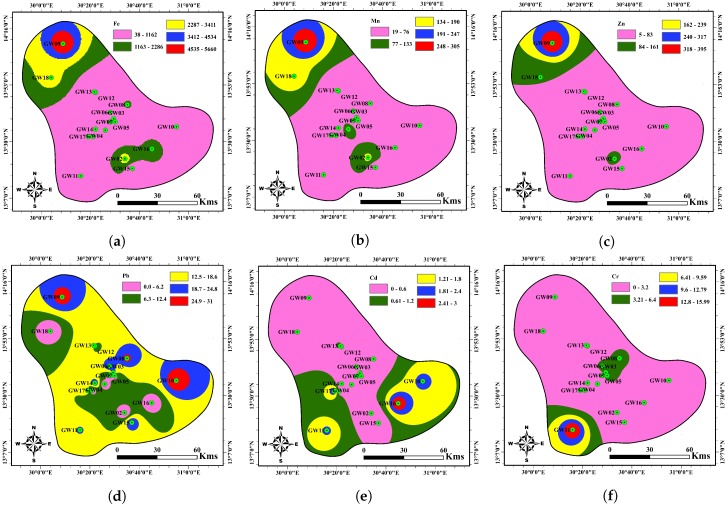
Spatial distribution of heavy metal ions in the groundwater samples and GIS interpolation maps of (**a**) Fe, (**b**) Mn, (**c**) Zn, (**d**) Pb, (**e**) Cd and (**f**) Cr in the study area.

Six heavy metal ions are considered in the present study to assess groundwater quality for drinking purposes. These elements in groundwater samples are assessed and compared against corresponding maximum permissible values of SSMO and WHO [27,28] standards for drinking water (Table 3). Table 3 also discusses the statistical behavior of each heavy metal in the study background, with their corresponding percentages exceeding the guideline values. The results showed that all heavy metals ions are within permissible limits of both standards except Fe in the samples obtained from Eltogoor, Um Balagie, Kewaimat, Um Nabag, Medaisis, Um Sout and Abu Shouk sites. Figure 2 shows the spatial distribution of sampling locations and GIS interpolation of heavy metal ions.

#### 4.1.1. Fe, Mn, Zn

Iron is a natural element of the earth’s crust [21]. It is present in groundwater due to the transfer of rainwater through the different earth layers and its friction with the elements of the soil that are saturated with quantities of iron [21,31]. The Fe concentration is over the MAC in most of the sampling sites. Most of the samples in the study area have a manganese level below MAC. Only at seven samples (GW02, GW04, GW09, GW10, GW11, GW17, and GW18), the concentration is above the MAC. The exceedance may be a result of over-pumping, which leads to the weathering of rocks containing manganese. In the study area, Zn concentration is below the MAC in all of the sampling sites.

#### 4.1.2. Pb, Cd, Cr

Pb, Cd, and Cr are toxic elements, and hazardous towards living beings [21,31]. The concentration of lead in the study area falls above the MAC in most of the samples (10 samples). The concentration of cadmium in the study area falls below the MAC in most of the samples (13 samples). All of the samples in the study area have a chromium level below MAC except the sampling site (GW11). The slight excedance of the three elements on the MAC value can be from natural sources.

### 4.2. Calculation of HPI, MI, PoS, and MHEI Indices

The models are applied to six heavy metals ions: Fe, Mn, Zn, Pb, Cd, and Cr to calculate the HPI based on Equations (1)–(4), MI using Equation (5), PoS using Equations (6)–(8) and MHEI, according to Equations (9)–(14). For the sake of limited space in this paper, the result of HPI for site GW01 is summarized in Table 4 as an example. Similarly, the computational results of MI, PoS, and MHEI are listed in Table 5, Table 6 and Table 7, respectively, for site GW01 as an example. Please note that the full-length results can be made available upon request. Regarding MHEI, we considered unit weights ($W_{i}$) for all heavy metal ions are inversely proportional to their MAC value (Equation (12)). SSMO [27] and (GB/T14848-1993) [29] of drinking water standards are used for calculating HPI, MI, PoS, and MHEI (Table 7). Table 7 summarizes the results of the proposed MHEI and compared methods.

The results of HPI (Table 8) show that all the groundwater samples in the study area are suitable for drinking purposes. The water quality classification according to the MI approach suggests that two samples are of very pure quality, seven samples are pure quality, six samples as slightly affected, two samples moderately polluted, and one sample seriously affected by the heavy metals. With respect to PoS, the results illustrate that the majority of groundwater samples (eight) show minimum, followed by five samples of low, three samples of a medium, and one sample of high-quality deterioration, respectively. The remaining sample falls into the category of severe deterioration. Regarding MHEI, the results show that one sample is of excellent quality, and thirteen samples are of good quality while the remaining samples are moderate water quality.

Specifically, 12 out of 18 samples have their NEI and PEI varying between −81 to −99 and 0.00 to 14.21, respectively, indicating excellent-good water quality. Also, two samples obtained PEI and NEI in the range of 27.73 to 28.84 and −0.03 to −8.2, respectively, signifying good water quality. The four remaining samples have PEI and NEI within the fields of 54.66 to 84.33 and 0.00 to −16.32, respectively, which indicates slightly polluted water.

### 4.3. Comparison of Indices Results

Table 8 shows the classifications of the groundwater samples based on MI, HPI, PoS, and MHEI. For the groundwater samples, all the heavy metals in it are below the permissible limit except iron in some samples. Through Table 8, Table 9 and Table 10, it is observed that neither HPI, PoS nor MI could judge the worthiness of water which MHEI could do easily. The best groundwater samples based on MI, HPI, PoS and MHEI indices are GW05, GW07, GW05, and GW05, respectively. In fact, any sample with PEI = 0 (all heavy metal ions are less than or equal to the highest desirable limit) is excellent water for drinking (e.g., GW05). Therefore, GW05 is the best as rightly indicated by the proposed MHEI, PoS, and MI. However, according to HPI, GW05 is 2nd best sample, which confirms the weakness of this indexing method. Also, the worst samples according to MI, HPI, PoS, and MHEI are GW09, GW16, GW09, and GW16, respectively. Concentrations of heavy metals (μg/L) in the best and worst groundwater samples are presented in Table 10. Despite increased concentrations of Fe, Mn, Zn, and Pb in sample GW09 as compared to sample GW16, the high-level concentration of Cd in sample GW16 dominates the effects of other heavy metals, and high toxicity of cadmium makes GW09 in the other order of the worst ranking [32].

MI and PoS consider the amount of concentration more than its importance in water quality. Also, regarding MI, sample GW09 is classified as seriously contaminated by heavy metals (i.e., MI > 6.0), but only the concentration of iron is higher than the permissible limit. The fact that the concentration of iron exceeds the permissible limit cannot justify the classification by MI and PoS for GW09. The sub negative and positive evaluation indices are the strengths of the proposed MHEI indexing method, which are missing in HPI, MI, and PoS indexing systems.

**Figure 3:**
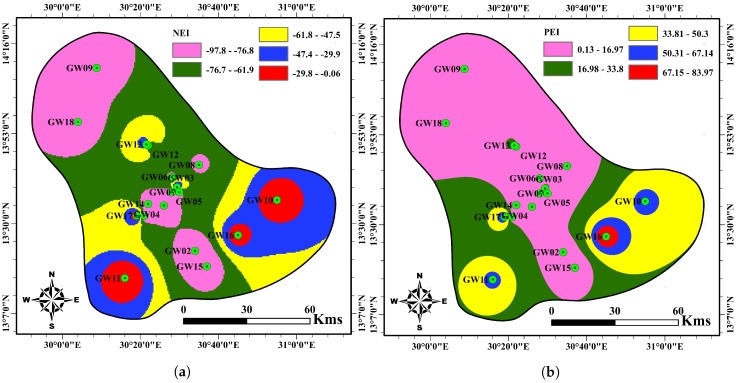
Spatial distribution of groundwater samples and GIS interpolation maps of MHEI (**a**) NEI and (**b**) PEI values in the study area.

**Figure 4:**
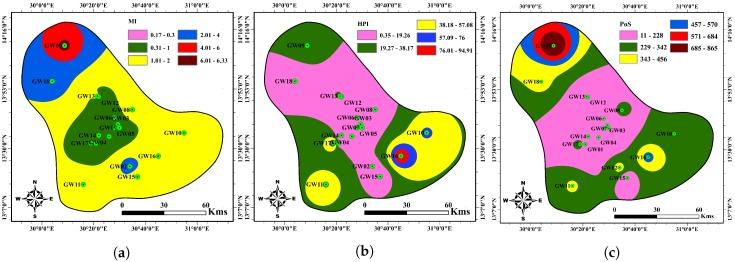
Spatial distribution of groundwater samples and GIS interpolation maps of (**a**) MI, (**b**) HPI and (**c**) PoS values in the study area.

Computed HPI and MHEI show that all groundwater samples from the boreholes are suitable for human consumption. The spatial distribution of NEI and PEI are in complete agreement with the metals spatial distribution (Figure 2 and Figure 3). Also, Figure 4 presents the spatial distribution of MI, HPI and PoS values for water quality in the study area. It is seen from Figure 3 and Figure 4 that the groundwater quality is good in most study areas, while a poor groundwater quality exists in the southeastern and southwestern parts.

## 5. Conclusions

The present study proposed a new method for heavy metals pollution indexing to assess groundwater quality for drinking purposes in North Kurdufan state, Sudan. The proposed method applies two indices in the water quality evaluation process; negative evaluation index (NEI) and positive evaluation index (PEI). The water quality is evaluated based on a pair of indices, NEI, and PEI. NEI and PEI directly reflect water quality: water quality increases with the decrease of PEI and NEI values. NEI indicates the contribution of heavy metals with a concentration not exceeding the highest desirable limit in water sample quality, while vice versa regarding the PEI. NEI varies from 0 to −100 while PEI may vary from 0 to any positive value. Eighteen groundwater samples collected from a confined aquifer have been analyzed. The results of MI, HPI, PoS and MHEI show that all groundwater samples from the boreholes are suitable for human consumption except sample (GW09) was found unsuitable as per MI and PoS methods. The proposed MHEI was compared with HPI, MI, and PoS which are widely used in the literature, and the following conclusions are drawn;

1. The spatial distribution of NEI and PEI are in complete agreement with the metals spatial distribution.
2. The MI and HPI indexing failed to account for the toxicity of elements in the evaluation of groundwater quality. This may explain why some samples were erroneously indexed. The proposed MHEI considered the element concentration as well as the toxicity in the groundwater quality evaluation dprocess to index the metals thoroughly, thus producing relatively better results.
3. The traditional heavy metals indexing techniques, namely MI, HPI, and PoS always take the metal concentration in water sample as a positive pollutant, even when the measured concentration is below the highest desirable limit. However, according to MHEI, heavy metal effect may be measured by a pair of indices, NEI and PEI. Additionally, some major shortcomings in the formulations of conventional indexing methods.
4. This study also proposed a more flexible water quality rating system that is more in sync with the standard guidelines documents.
5. The performance of the MHEI model proposed was strong, promising, and proved useful for evaluating heavy metals pollution levels in groundwater. It also takes care of many deficiencies of the existing approaches.

The proposed MHEI indexing can provide accurate and reliable information on water quality and serves as a useful tool for sustainable water resource management in the future.

## Figures and Tables

**Table 1:** The classification of water quality degradation level according to PoS index.

Toxic Class	Parameters	Points	P-Class	*w* ${}_{i}$	*W* ${}_{i}$	Quality Degradation
5	Pb	10	V	8	0.301887	High
5	Cd	10	V	8	0.301887	
4	Cr	100	IV	5	0.188679	Moderate
3	Zn	1000	III	3	0.113208	Non-low
2	Mn	5000	II	1.5	0.056604	
1	Fe	50,000	I	1	0.037736	

**Table 2:** Water quality classification based on the MHEI.

MHEI Value Range	Measured Concentration Range	Type of Water
−100 ≤ NEI ≤ 0 and PEI = 0	DL ≥ $M_{i}$ ≤ $I_{i}$	Excellent water
−100 < NEI ≤ 0 and 0 < PEI ≤ 50	$I_{i}$ < $M_{i}$ ≤ $S_{i}$	Good water
−100 < NEI ≤ 0 and 50 < PEI ≤ 100	$I_{i}$ < $M_{i}$ ≤ $S_{i}$	Moderate water
−100 < NEI ≤ 0 and PEI = 50	$I_{i}$ < $M_{i}$ ≤ $S_{i}$	Poor water
NEI = 0 and PEI > 100	$M_{i}$ > $S_{i}$	Water unsuitable for drinking purposes

**Table 3:** Descriptive statistics of heavy metal ions in the groundwater samples and their comparison with SSMO (2002) and WHO (2011) guideline values for drinking water.

Parameters	${}^{a}$ Min.	${}^{b}$ Max.	Mean	Median	${}^{c}$ Std. Dev	SSMO (2002)	WHO (2011)	% Exceeding Guideline Value
Fe	36.8	5661.4	956.99	220.3	1435.37	300	300	38.89
Mn	19.9	304.9	75.37	43.85	74.07	500	400	0.00
Zn	5.4	395.0	53.49	21.3	88.2	3000	3000	0.00
Pb	${}^{d}$ ND	31.0	25.09	26.95	4.22	100	100	0.00
Cd	ND	3.0	1.93	1.95	0.76	3.0	3.0	0.00
Cr	ND	16.0	7.06	5.0	4.58	40	50	0.00

Note: All heavy metals ions unit in μg/L, ${}^{a}$ Mminimum, ${}^{b}$ Maximum, ${}^{c}$ Standard Deviation, and ${}^{d}$ Not Detected.

**Table 4:** The $W_{i}$, $Q_{i}$, and HPI calculation for groundwater samples (GW01 as an example) of the study area.

Parameters	${}^{a}$ *M* ${}_{i}$	${}^{b}$ *S* ${}_{i}$	${}^{c}$ *I* ${}_{i}$	${}^{d}$ *w* ${}_{i}$	*^e^ W* ${}_{i}$	${}^{f}$ *Q* ${}_{i}$	*W*${}_{i}$ × *Q*${}_{i}$
Fe	288.5	300	100	0.0033	0.009	94.25	0.840
Mn	26.0	500	50	0.0020	0.005	5.333	0.029
Zn	21.3	3000	50	0.0003	0.001	0.973	0.001
Pb	ND	3100	5.0	0.0100	0.027	0.00	0.00
Cd	ND	3.0	0.1	0.3333	0.891	0.00	0.00
Cr	ND	40	5.0	0.0250	0.067	0.00	0.00
HPI = $\sum_{i=1}^{n}$$W_{i}$$Q_{i}$							0.87

${}^{a}$ Measured concentration value, ${}^{b}$ Maximum permissible value, ${}^{c}$ Highest desirable value, ${}^{d}$ Unit weight factor, *^e^* Relative weight, and ${}^{f}$ Sub index.

**Table 5:** The MI calculation for groundwater samples (GW01 as an example) of the study area.

Parameters	*M* ${}_{i}$	${}^{a}$ MAC${}_{i}$	*M*${}_{i}$/MAC${}_{i}$
Fe	288.5	200	1.443
Mn	26.0	50	0.520
Zn	21.3	500	0.043
Pb	ND	10	0.00
Cd	ND	1.0	0.00
Cr	ND	10	0.00
MI = Mean value			0.334

${}^{a}$ Maximum allowable value.

**Table 6:** The PoS calculation for groundwater samples (GW01 as an example) of the study area.

Parameters	*M* ${}_{i}$	*S* ${}_{i}$	*w* ${}_{i}$	*W* ${}_{i}$	*Q* ${}_{i}$
Fe	288.5	300	1	0.037736	36.29
Mn	26.0	500	1.5	0.056604	2.94
Zn	21.3	3000	3	0.113208	0.80
Pb	ND	3100	8	0.301887	0.00
Cd	ND	3.0	8	0.301887	0.00
Cr	ND	40	5	0.188679	0.00
PoS = PoS = Aggregation of all $Q_{i}$					40

**Table 7:** Standard values of corresponding heavy metals ions and calculation of MHEI for groundwater samples (GW01 as an example) of the study area.

Parameters	*M* ${}_{i}$	*S* ${}_{i}$	*I* ${}_{i}$	MAC${}_{i}$	${}^{a}$ *W* ${}_{i}$	${}^{b}$ ω ${}_{i}$	*Q* ${}_{i}$	ω${}_{i}$ × *Q*${}_{i}$	${}^{c}$ PEI	${}^{d}$ NEI
Fe	288.5	300	100	200	0.005	0.004	94.25	0.38	0.38	−97.89
Mn	26.0	500	50	50	0.020	0.016	−5.33	−0.09		
Zn	21.3	3000	50	500	0.002	0.002	−0.97	0.00		
Pb	ND	100	5.0	10	0.100	0.082	−100	−8.15		
Cd	ND	3.0	0.1	1.0	1.000	0.815	−100	−81.5		
Cr	ND	40	5.0	10	0.100	0.082	−100	−8.5		

${}^{a}$ Unit weight factor, ${}^{b}$ Relative weight, ${}^{c}$ Positive evaluation index, and ${}^{d}$ Negative evaluation index.

**Table 8:** Comparison results of water quality classification by MI, HPI, PoS, and MHEI.

ID	Location	MI		HPI		PoS			MHEI		
		Score	Class	Score	Class	Score	${}^{a}$ Dominant	Class	PEI	NEI	Class
GW01	Um Galagie	0.33	Pure	0.87	Suitable for drinking purposes	40	Fe	Minimum	0.38	−97.89	Good
GW02	Eltogoor	2.97	Moderately	12.61		387	Fe, Mn	Medium	6.08	−97.80	Good
GW03	Hamdan 1	1.03	Slightly	28.95		242	Pb	Low	27.73	−0.03	Good
GW04	Hamdan 2	0.67	Pure	0.31		37	Mn	Minimum	0.49	−97.80	Good
GW05	Um Ushar 1	0.16	Very pure	0.27		10	-	Minimum	0.00	−98.26	Excellent
GW06	Um Ushar 2	0.71	Pure	0.90		116	Pb	Minimum	1.89	−81.87	Good
GW07	Um Laham	0.31	Pure	0.24		46	-	Minimum	0.10	−89.72	Good
GW08	Um Balagie	1.79	Slightly	6.27		274	Fe, Pb	Low	4.38	−81.75	Good
GW09	Kewaimat	6.33	Seriously	25.73		845	Fe, Mn, Pb	Severe	14.21	−89.65	Good
GW10	Um Samima	1.21	Slightly	59.58		327	Mn, Pb, Cd	Low	55.85	−8.15	Moderate
GW11	Um Gewaiz	1.30	Slightly	58.45		362	Fe, Mn, Pb, Cd, Cr	Medium	54.66	0.00	Moderate
GW12	Elhadid	0.25	Very pure	0.44		27	-	Minimum	0.19	−97.89	Good
GW13	NUm Nabag	1.52	Slightly	31.90		303	Fe	Low	28.84	−8.20	Good
GW14	Bara	0.55	Pure	0.74		88	Pb	Minimum	1.75	−90.71	Good
GW15	Medaisis	0.96	Pure	2.41		142	Fe, Pb	Minimum	2.46	−89.70	Good
GW16	Namil	1.90	Slightly	95.31		495	Fe, Cd	High	84.33	−16.32	Moderate
GW17	Um Sout	0.96	Pure	80.52		332	Mn, Pb, Cd	Low	74.06	−8.56	Moderate
GW18	Abu Shouk	2.96	Moderately	12.10		374	Fe, Mn	Medium	5.95	−97.80	Good

${}^{a}$ The score of heavy metal is above the threshold value.

**Table 9:** Best and worst groundwater samples based on MI, HPI, PoS, and MHEI.

Sample Rank	MI		HPI		PoS		MHEI		
	ID	Value	ID	Value	ID	Value	ID	PEI	NEI
Best	GW05	0.16	GW07	0.24	GW05	10	GW05	0.00	−98.3
Worst	GW09	6.33	GW16	95.31	GW09	845	GW16	84.3	−16.3

**Table 10:** Concentrations of heavy metal ions (μg/L) in the best and worst groundwater samples in the study area.

Sample Rank	ID	Fe	Mn	Zn	Pb	Cd	Cr
Best	GW05	44.8	34.7	21	ND	ND	ND
	GW07	48.6	29.8	19.4	ND	ND	5.0
Worst	GW09	5661.4	304.9	395	27.6	ND	ND
	GW16	1487.3	45.9	12	ND	3.0	ND
